# Deferasirox and Vitamin D Improves Overall Survival in Elderly Patients with Acute Myeloid Leukemia after Demethylating Agents Failure

**DOI:** 10.1371/journal.pone.0065998

**Published:** 2013-06-20

**Authors:** Etienne Paubelle, Florence Zylbersztejn, Sawsaneh Alkhaeir, Felipe Suarez, Céline Callens, Michaël Dussiot, Françoise Isnard, Marie-Thérèse Rubio, Gandhi Damaj, Norbert-Claude Gorin, Jean-Pierre Marolleau, Renato C. Monteiro, Ivan C. Moura, Olivier Hermine

**Affiliations:** 1 CNRS UMR 8147, Université Paris Descartes, Faculté de Médecine, Hôpital Necker, Paris, France; 2 INSERM U699, Paris, France; 3 Insitut Hospitalo-Universitaire (IHU) Imagine, Université Sorbonne Paris cité, Assistance Publique-Hôpitaux de Paris, Hôpital Necker, Paris, France; 4 Service d’Hématologie clinique, Assistance Publique-Hôpitaux de Paris, Hôpital Necker, Paris, France; 5 Laboratoire d’excellence des Globules Rouges (GR-ex), Paris, France; 6 Department of Hematology, AP-HP Hôpital Saint Antoine, Université Pierre et Marie Curie, Paris, France; 7 Centre Hospitalier Universitaire (CHU) Sud, Amiens, France; 8 Service d’Immunologie, Hôpital Bichat, Assistance Publique Hôpitaux de Paris, Paris, France; 9 Faculté de Médecine and Université Denis Diderot Paris VII, Paris, France; University of Saarland Medical School, Germany

## Abstract

The prognosis of acute myeloid leukemia (AML) in elderly (≥65 years) patients is poor and treatment remains non-consensual especially for those who are not eligible for intensive therapies. Our group has shown that *in vitro* the iron chelator deferasirox (DFX) synergizes with vitamin D (VD) to promote monocyte differentiation in primary AML cells. Herein, we present results from a retrospective case-control study in which the association of DFX (1–2 g/d) and 25-hydroxycholecalciferol (100,000 IU/week) (DFX/VD) was proposed to patients following demethylating agents failure. Median survival of patients treated with DFX/VD combination (n = 17) was significantly increased in comparison with matched patients receiving best supportive care (BSC) alone (n = 13) (10.4 versus 4 months respectively). In addition, the only factor associated to an increased overall survival in DFX/VD-treated patients was serum VD levels. We conclude that DFX/VD treatment correlated with increased overall survival of AML patients in this retrospective cohort of elderly patients.

## Introduction

Acute myeloid leukemia (AML) is a group of heterogeneous malignant diseases characterized by uncontrolled cell growth and differentiation arrest [1]. Prognosis of older patients diagnosed with AML is particularly poor and almost did not change in the last decades [2]. Although, 40% to 60% of them achieve a complete remission (CR) following with intensive chemotherapy [3], the overall survival for this group of patients (cut off ranging from 60 to 70 years) is between 4 to 7 months [4]–[6] compared to approximately 20 months for the entire population of patients with AML [1]. Recently it has been shown that the use of demethylating agents (such as 5-azacytidine or decitabine) may induce hematological responses and increase life expectancy in elderly patients [7]. However, management of patients following epigenetic therapy failure is not consensual and best supportive care (BSC) is still proposed for most of these patients. Thus, new therapeutic strategies are strongly needed in this population of AML patients unfit or relapsing/refractory to high dose chemotherapy.

Following the success of differentiating therapies in acute promyelocytic leukemia (APL), great hopes were placed in Vitamin D (VD) and its ability to promote monocyte differentiation of non-APL AML cells [8]. However, results of clinical studies were disappointing and trials were interrupted due to the occurrence of life-threatening hypercalcemia [9].

Most elderly patients diagnosed with AML exhibit secondary iron overload because of iterative red blood cell transfusions and, in some cases, an increased of iron absorption due to ineffective erythropoiesis [10]. Interestingly, in myelodysplastic syndromes (MDS), retrospective studies have suggested that iron chelators may increase life expectancy and may decrease the risk of transformation into AML although the molecular mechanism involved remains unknown [11], [12]. It has been shown that deferasirox, DFX is able to induce AML cells apoptosis *in vitro* through inhibition of NF-κB pathway [13]. In addition, our group has shown that *in vitro* iron chelators are able to promote monocyte differentiation in both normal hematopoietic progenitors and primary AML cells and that iron privation agents acted synergistically with VD to promote cell differentiation [14]. Because of cumulated evidences from experimental data and that both iron chelators and VD analogues are safe drugs and currently used in AML patients, we proposed deferasirox and 25-hydroxycholecalciferol combination therapy to a 69-yr-old AML patient, who was refractory to high dose chemotherapy. DFX/VD treatment was associated with reduced blast counts and partial reversal of pancytopenia, accompanied by an increase in monocyte numbers derived from the blast pool [14]. This case-study suggested that the combined DFX/VD therapy could act as a differentiating agent in human AML. Taken together, these observations provided a strong rationale for the use of iron chelators/VD therapy in the setting of high risk or relapsing AML elderly patients after failure or not fit enough for chemotherapy.

Since VD deficiency and iron overload prevalence is high in the elderly AML patients, the association of VD and DFX was given to a subgroup of patients following demethylating agents failure. Here we report a case-control retrospective study (performed in three independent medical centers) aiming to investigate the therapeutic potential of the association of VD and DFX in elderly AML patients following demethylating agents failure. We show that patients who received this combination therapy presented an increased overall survival and that VD serum level was the stronger factor that positively correlated with overall survival.

## Methods

### Patients

A retrospective chart review of 17 elderly AML patients following demethylating agents failure was performed in three French centers. The combination of DFX/VD was proposed to patients who were not eligible to any other treatment. Matched controls (13 individuals) were patients receiving best supportive care (BSC) during the same period. There were no differences in blood tests panels or AML prognostic factors between combination therapy and BSC (data not shown). In each case, the dose of DFX was adapted based on ferritin and creatinine levels. DFX initial dose was of 20–30 mg/kg per day and VD was used at 100,000 units orally weekly.

### Ethics Statement

This study received ethics approval from the Human Ethics Committee of the Necker hospital (IRB registration #00001072). According to French legislation no written consents are necessary for observational retrospective studies. All data were analyzed anonymously. Each patient was identified with a personal number. Patients were aware that their data were stored in a specific database, but were not informed that these data were used for research purposes. This procedure has been disclosed to the Ethics Committee that, in accordance with national legislation, approved it.

### Statistical Analyses

Statistical analyses were performed with Graphpad Prism® and SAS JMP8®. Χ^2^ statistic and Fisher exact test for discrete variables were used to compare proportions, and Student *t* test was used for continuous variables. The onset of treatment was considered for both groups as the time from which patients had stopped to receive 5-azacytidine treatment. Overall survival (OS) was defined as the time from the initiation of therapy to death from any cause and was censored at the date of last information. Survival was estimated by the method of Kaplan-Meier and was compared by the use of the log-rank test. Multivariate analysis was performed using Cox proportional hazard model. Statistical significance was defined as p-value <0.05 (*), <0.01 (**), or <0.001 (***).

## Results and Discussion

In our retrospective case-control study the median age was not statistically different between the DFX/VD (76 years; range, 63–84) and BSC patients (71 years; range 58–85) groups (Table 1). Most patients were diagnosed with AML with multilineage dysplasia (DFX/VD cases 70%, BSC controls 76%) and cytogenetic prognosis groups were distributed homogeneously between the patients and controls (Table 1). In addition, there were no significant differences in blast infiltration, leukocytosis, neutropenia, systemic iron and phospho-calcium parameters (Table 1). All patients received 5-azacytidine (median of 8 and 7 courses for the cases and the controls, respectively).

**Table 1:** Table **1.** Baseline characteristics of patients.

Characteristics	Cases	Control	p
	median (range)	Median (range)	
age (years)	76 (63–84)	71 (58–85)	ns
*sex ratio* (M/F; %)	41	46	ns
Number of 5-azacytidine courses	8 (3–24)	7 (2–18)	ns
AML II or AML with multilineage dysplasia (%)	70	76	ns
leukocytes (G.L^−1^)	0.3 (0–55)	3.8 (0.8–34)	ns
granulocytes (G.L^−1^)	0.1 (0–5.6)	0.8 (0.3–6)	ns
monocytes (G.L^−1^)	0 (0–1.8)	0.1 (0–2.4)	ns
blood blast level (%)	15 (1–87)	23 (0–83)	ns
medullary blast count (%)	26 (4–71)	22 (16–54)	ns
favorable karyotype (%)	0	0	
intermediate karyotype (%)	82	84	ns
adverse risk karyotype (%)	18	15	ns
ferritin (µg.L^−1^)	1412 (1310–1850)	1500 (1100–1910)	ns
creatinine (µmol.L^−1^)	67 (50–124)	71 (60–139)	ns
calcium (mmol.L^−1^)	2.21 (2.12–2.36)	2.10 (1.95–2.24)	ns
phosphates (mmol.L^−1^)	1.18 (0.91–1.43)	1.16 (0.96–1.37)	ns
vitamin D (25-OH; nmol.L^−1^)	33.5 (5.5–117)	37	nd
proteins (g.L^−1^)	68 (65–75)	70 (60–74)	ns

**Figure 1:**
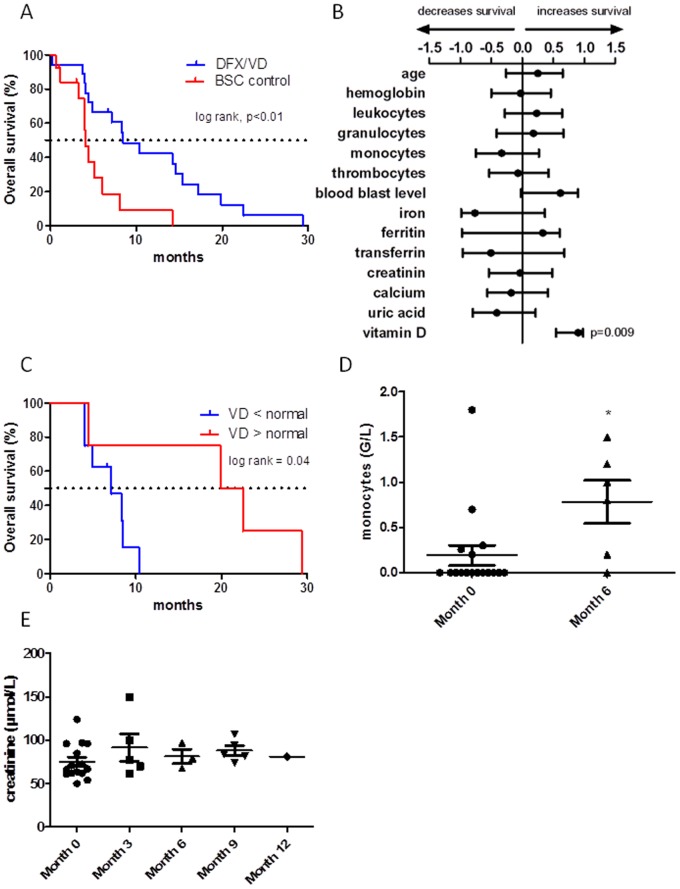
DFX/VD association induces myeloid differentiation and increases overall survival in elderly AML patients. (A) Kaplan-Meier estimated OS in DFX/VD and BSC treated patients (B) Multivariate analysis. Forest plot of the odds ratio. (C) OS within subgroups presenting normal VD levels (≥50 nmol/L) or VD deficiency (≤50 nmol/L). (D) Monocytes numbers in VD/DFX treated patients (F) Creatinine levels in treated patients.

When evaluating patients’ outcome between the two groups we observed that median survival of treated patients was significantly increased relative to BSC group (10.4 months versus 4 months, p = 0.002) (figure 1A). Multivariate analysis realized in the DFX/VD group showed that among the several blood parameters evaluated only serum levels of VD prior to treatment was able to predict patients’ outcome (figure 1B). Patients with normal VD levels (≥50 nmol/L) had a significant increase of median OS compared to the group of VD deficiency (≤50 nmol/L) (21.2 versus 7.1 months respectively; p = 0.04) (figure 1C). Serum VD levels were not correlated to nutritional status (data not shown).

By evaluating hematological parameters at 6 months, we observed that 4 treated patients (out of 17 still alive at this time point; 23.5%) had significant increased monocyte numbers (figure 1D). The treatment did not decrease the need of transfusion in both VD/DFX and BSC groups. Serum creatinine levels did not incresead following DFX/VD therapy (figure 1E) and hypercalcemia or hepatotoxicity (data not shown) were not observed suggesting the safety of the combined therapy in this population of patients.

Kantarjian *et al* have shown that in elderly patients AML, OS was of 4 months [6]. Furthermore, in a recent study that reviewed the outcome of patients with newly-diagnosed AML aged 65 or older treated with demethylating agents CR rate was of 28% and median survival of only 6.5 months. Moreover, no difference in the outcome was observed according to prognosis subgroups (cytogenetics, FLT3 mutational status, age…) [15]. Here, we show that the DFX/VD differentiating therapy has a beneficial effect in elderly AML patients following demethylating agents failure. Although no patient has achieved CR, we observed that the DFX/VD association was able to improve drastically OS without toxicity. BSC patients were similar with respect to clinical and biological parameters and exhibited poor OS, which was comparable to the reported in the literature [6]. The elevation of monocyte levels suggest that this increase in overall survival might be due to a differentiating effect promoted by the use of the combined therapy. In a recent study, Nasr *et al* have shown that arsenic trioxide and retinoids, in addition of their differentiating effect, were able to affect leukemic initiating cells (LIC) [16]. Therefore, further studies should evaluate the impact of DFX/VD on LIC. These encouraging results from this retrospective study should, however, be verified in a large randomized prospective multicenter study.
